# How a smiley protects health: A pilot intervention to improve hand hygiene in hospitals by activating injunctive norms through emoticons

**DOI:** 10.1371/journal.pone.0197465

**Published:** 2018-05-21

**Authors:** Susanne Gaube, Dimitrios Tsivrikos, Daniel Dollinger, Eva Lermer

**Affiliations:** 1 Department of Experimental Psychology, University of Regensburg, Regensburg, Germany; 2 Division of Psychology and Language Sciences, University College London, London, United Kingdom; 3 Institute of Flight System Dynamics, Technical University of Munich, Garching bei München, Germany; 4 FOM University of Applied Sciences, Munich, Germany; Mälardalen University, SWEDEN

## Abstract

Hand hygiene practice in hospitals is unfortunately still widely insufficient, even though it is known that transmitting pathogens via hands is the leading cause of healthcare-associated infections. Previous research has shown that improving knowledge, providing feedback on past behaviour and targeting social norms are promising approaches to improve hand hygiene practices. The present field experiment was designed to direct people on when to perform hand hygiene and prevent forgetfulness. This intervention is the first to examine the effect of inducing injunctive social norms via an emoticon-based feedback system on hand hygiene behaviour. Electronic monitoring and feedback devices were installed in hospital patient rooms on top of hand-rub dispensers, next to the doorway, for a period of 17 weeks. In the emoticon condition, screens at the devices activated whenever a person entered or exited the room. Before using the alcohol-based hand-rub dispenser, a frowny face was displayed, indicating that hand hygiene should be performed. If the dispenser was subsequently used, this picture changed to a smiley face to positively reinforce the correct behaviour. Hand hygiene behaviour in the emoticon rooms significantly outperformed the behaviour in three other tested conditions. The strong effect in this field experiment indicates that activating injunctive norms may be a promising approach to improve hand hygiene behaviour. Theoretical and practical implications of these findings are discussed.

## Introduction

Healthcare-associated infections are a rising threat to global health systems. Infections acquired while receiving healthcare can cause severe impairments to patients’ physical and mental health, increase death rates, and add massive costs to health systems [1,2]. According to one estimate, over four million patients suffer from healthcare-associated infections in Europe annually. Of these cases, approximately 37,000 patients die as a direct consequence [3]. Due to the rise of antimicrobial resistant organisms, this number is likely to increase in the future [4]. Contact with contaminated healthcare workers’ hands has long been known as the main vehicle for transmitting pathogens that cause healthcare-associated infections [2]. In addition, patients’ and visitors’ hands have been recently identified as a severe risk factor for the spread of germs [5,6].

Improving hand hygiene has proven to be an effective method of decreasing infection rates, as well as the spread of antimicrobial resistant organisms [7–9]. However, hand hygiene practice in healthcare is still largely insufficient. On average, healthcare personnel in industrialised countries around the world clean their hands in only 40% of the cases required by guidelines [10]. Patients’ and visitors’ hand hygiene behaviour is estimated to be even lower [11,12].

### Psychological factors influencing hand hygiene behaviour

From a theoretical standpoint, Ajzen’s theory of planned behaviour [13] is the most widely used model to explain hand hygiene behaviour [14]. The theory postulates that behaviour–in this case, hand hygiene–is driven by the *intention* to perform this action. The intention is predicted by three factors: first, *attitude*, which is formed by knowledge and beliefs about hand hygiene and its outcomes. Second, *subjective norms*, shaped by a person’s perception of how others think about hand hygiene. Third, *perceived behaviour control*, which is composed of beliefs about the ease or difficulty of performing hand hygiene [15]. Some researchers have reported positive correlations between all three factors and hand hygiene behaviour among healthcare personnel [16–19]. However, in other studies, not all factors correlated with hand hygiene [15,20,21]. No single variable emerged as being particularly influential above and beyond the others.

Scholars have indicated that focusing solely on one theory is too narrow of an approach [22,23]. Therefore, researchers have examined predictors for non-compliance with hand hygiene guidelines among healthcare workers within the theoretical domains framework, which is based on a range of behavioural theories [24]. They found three theoretical domains to predict more than three-quarters of non-compliance: *memory/attention/decision making* (i.e., forgetting, being distracted or prioritising another task); *knowledge* (i.e., lack of knowledge about guidelines); and *environmental context/resources* (i.e., lack of time or availability of products) [23].

To sum everything up, several variables have been identified as predictors for hand hygiene behaviour in hospitals. The quintessence of the research suggests the following: 1) Hand hygiene products need to be available; 2) People need to know when to perform hand hygiene; 3) The environment should be designed to prevent forgetfulness and support hand hygiene behaviour; and 4) Social norms should encourage hand hygiene behaviour. Alcohol-based hand-rub products–the gold-standard for hand hygiene–are widely accessible in Western hospitals [25]. However, further research is needed to establish a solid theoretical foundation concerning the timing of hand hygiene behaviour, environmental design, and the role of social norms.

### Previous interventions to improve hand hygiene behaviour

Numerous interventions have been previously implemented to increase hand hygiene compliance with guidelines among healthcare personnel [26–33]. This volume of literature signifies the importance, but also the difficulties, associated with finding approaches to shift behaviour in this domain. One challenge with interventions is that they only modestly improve hand hygiene practice. For instance, relying solely on signs and posters seems to have no sizeable effect on improving hand hygiene behaviour [34,35]. Even when an intervention substantially increases compliance rates, the positive effect often lasts only for a short period of time. This is especially true after on-off education-based programmes, where compliance rates have been found to fall back to pre-intervention levels [34,36]. However, previous studies targeting the three aforementioned factors (i.e., knowledge of when to perform hand hygiene, reminders to prevent forgetfulness, and social norms) have shown promising results.

One intervention to improve knowledge via reoccurring, goal-oriented and reinforcing personal feedback, led to significantly higher hand hygiene compliance over three years compared to a control condition [37]. However, the programme was labour-intensive, which negatively affected implementation. Automated electronic monitoring with feedback systems has been suggested as an alternative to direct observation and personal feedback, as this approach decreases workload and eliminates observation biases [32]. The main benefit is that these systems can indicate when to perform hand hygiene at all times. One study used visual and auditory prompts at alcohol-based hand-rub dispensers, as a reminder to perform hand hygiene whenever someone passed by [38]. While the outcomes were promising, additional evidence is needed to verify the study’s results. More advanced systems have multiple sensors in the patient area to monitor hand hygiene opportunities. Here, healthcare workers need to wear badges, which register hand hygiene opportunities and events (i.e., using an alcohol-based hand-rub dispenser). The badges operate as indicators of when to perform hand hygiene, providing real-time feedback about hand hygiene status via light [39,40] or sound [41]. The greatest benefit of extensive electronic monitoring systems is that they offer constant, non-punitive, and direct feedback, which has been shown to be the most effective form of feedback [42]. However, such systems have limitations: 1) Wireless systems might interfere with medical equipment; 2) The systems are expensive and need maintenance; and 3) Healthcare workers are not comfortable with being constantly monitored [43]. Results of the effects of extensive systems on hand hygiene compliance have been mixed but promising [43]. Overall, interventions aimed to increase knowledge on when to perform hand hygiene via reminders and feedback have been shown to improve hand hygiene behaviour in hospitals. However, all attempts showed various limitations, and there is still room for improvement. As these systems are very expensive, it should be sufficiently tested which exact factors have a positive effect on hand hygiene behaviour prior to their implementation.

Previous research has also successfully targeted social norms to improve hand hygiene behaviour in hospitals. One study found that adding social norm elements (e.g., setting team norms and role modelling) to a traditional intervention (including education, reminders and group feedback) further improved hand hygiene compliance among hospital workers [44]. However, this approach is labour intensive, which makes it hard to uphold over time. Another intervention used a remote video auditing system to improve hand hygiene compliance by publicly sharing performance feedback [45]. The adherence rate increased from 6.5% to 81.6%, but the intervention was expensive because of the cost associated with camera installation and the ongoing evaluation of video footage. Additionally, video surveillance would not be acceptable in many countries and, as mentioned before, people feel uneasy about being constantly monitored.

Previous campaigns have typically used role modelling and peer pressure to activate social norms (e.g., [44]). However, there might be less labour-intensive ways to target social norms. Research has shown that displaying images of eyes or eye-like illustrations increases pro-social behaviour through the feeling of being observed. This phenomenon is known as the *watching eyes effect*, and it has been found in lab-based studies (e.g., [46]) as well as in field experiments (e.g., [47,48]). However, it should be noted that a recently conducted meta-analysis examining the surveillance effect on generosity raised scepticism on its robustness [49]. Besides this, the watching eyes effect has been previously tested on hand hygiene behaviour; in a series of experiments, pictures of human eyes were placed at hand-rub dispensers in hospitals [50–52]. Two [50,52] out of the three studies found that dispensers with images of eyes were used more frequently than dispensers without such illustrations, while the other [51] found no significant difference. One possible, but controversially discussed, explanation for the watching eyes effect could be that eye cues make people more inclined to follow salient *local* or so-called *descriptive norms* (i.e., ‘I should do what everybody else is doing’) ([53,54] but see [47] for opposing view). This norm-based approach might explain some of the mixed findings in the hand hygiene studies, as descriptive norms could vary between healthcare facilities. Additionally, scholars have argued that utilising descriptive norms on their own can backfire, because descriptive norms can differ considerably from *injunctive norms* (i.e., ‘I should do, what ought to be done’) [55,56]. Therefore, if the eye-like image makes descriptive norms salient, and a healthcare professional or layperson thinks that most other people do not perform hand hygiene, they would be more likely to copy this bad behaviour to comply with the norm. A simple solution for this problem was offered in the context of energy consumption [56]: Using emoticons as a form of injunctive normative feedback to prevent the boomerang effect, since the images convey social disapproval (frowny face) or approval (smiley face) very clearly. Negatively and positively valanced emoticons have also been successfully used in behavioural change settings to reduce food waste [57], water consumption [58] and speeding [59]. However, to our knowledge, they have never been applied in interventions to improve hand hygiene behaviour in hospitals. In summary, utilising social norms to change behaviour has been shown to be successful, but relying on descriptive norms might backfire. Therefore, it should be investigated if targeting injunctive norms might prove to be a more reliable approach in this highly sensitive field.

### The present study

An electronic monitoring and feedback system was installed on top of alcohol-based hand-rub dispensers inside patient rooms in a German hospital, in order to measure room traffic and display visual cues. Only entrances and exits were monitored via motion sensors, which has been shown to capture around 80% to 85% of all possible hand hygiene opportunities in patient care [43]. This has three main benefits: First, it is an adequate but cost-efficient approach compared to more extensive systems. Second, healthcare staff find entrance/exit monitoring more acceptable than being under constant surveillance [60]. Third, it includes patients and visitors in addition to healthcare personnel, as recommended by German guidelines for hand hygiene in healthcare facilities [25]. The last point is a strong advantage of the present study, as many previous interventions only targeted healthcare professionals.

The objective of the present study was to test whether or not visual reminders with the potential to activate social norms affect hand hygiene behaviour in hospital patient rooms. Based on the literature reviewed, three research questions were formulated:

*Research question 1*: Does the display of emoticons, which is assumed to induce injunctive norms by providing instant feedback about current hand hygiene status, improve hand hygiene behaviour in patient rooms?

*Research question 2*: Can images of human eyes, which potentially make descriptive norms salient, improve hand hygiene practice in patient rooms? (Replication of the watching eyes effect)

*Research question 3*: Are visual cues displaying emoticons more effective than visual cues displaying human eyes in changing hand hygiene behaviour?

## Materials and method

### Design

This field experiment was conducted at the Rottal Inn Hospital in Eggenfelden, Germany. In consultation with the hygiene specialists at the hospital, eight out of the sixteen patient rooms within the trauma surgery unit were randomly chosen. This unit was selected for the pilot intervention because it usually has the highest patient turnover rate, a diverse mix of patients (e.g., age, gender and disease patterns) and is a high-risk area for post-surgical infections. These rooms can be accessed by healthcare staff, patients and visitors. The eight rooms are nearly identical in design, with two patient beds and an ensuite bathroom. Each room only has one dispenser, but there is another one in the hallway next to each room’s entrance. Ethical approval for the study was obtained from the UCL Research Ethics Committee. Both the Ethics Committee and the hospital's management team waived the requirement for informed consent as the experiment was of a passive nature. At no point did we collect any personal data (e.g., names, gender) of patients and/or medical staff. Our experimental design and research protocol did not influence existing processes, necessary to allow the smooth operation of the hospital.

### Manipulations

**Fig 1:**
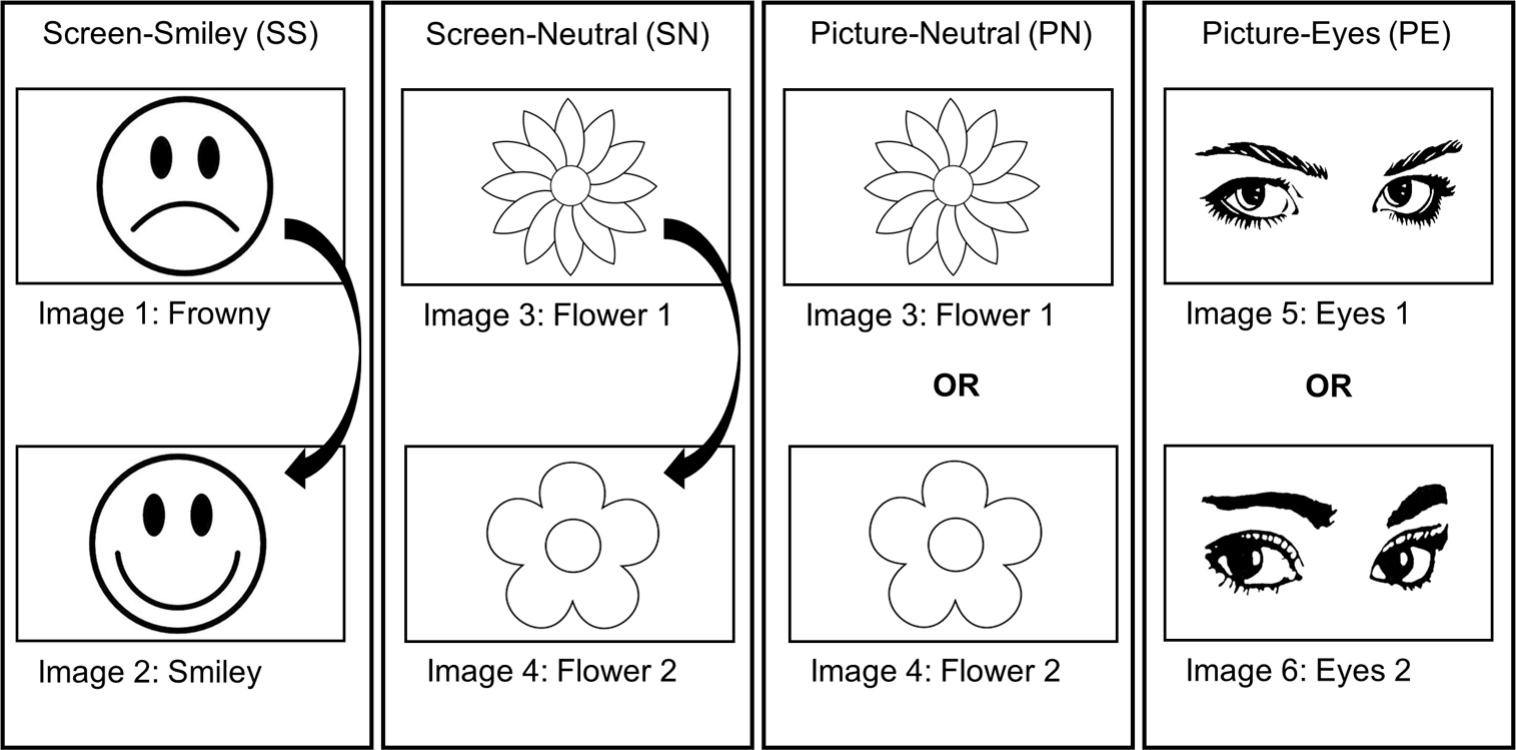
Experimental conditions and images used during the intervention phase.

This study had a baseline and an intervention phase. The latter consisted of four conditions (see Fig 1), which are described below. To aid discoverability and possible synthesis of the results with other interventions of health psychology studies, the relevant components of the Behaviour Change Technique Taxonomy v1 (BCT-T-v1) [61] are outlined within each condition description. 1) *Screen-Emoticons*: In the emoticon condition, the screen at the device turned on whenever the motion sensor was activated (i.e., when people entered or left the room), displaying a frowny face. The frowny turned into a smiley when the hand-rub dispenser was used. The frowny stayed activated for 25 seconds unless the dispenser was used, in which case the smiley was also displayed for 25 seconds. After either 25-second period, the screen switched off again. The Screen-Emoticons condition was expected to attract attention to the fact that hands should be cleaned at this point and prevent forgetfulness through visual cues (BCT-T-v1: prompts/cues). It was supposed to activate injunctive norms via the frowny face by providing direct feedback about the hand hygiene status for the user (wrong behaviour: sad face; right behaviour: happy face; BCT-T-v1: feedback on behaviour and social reward).

2) *Screen-Neutral*: In the first neutral control condition, the screen turned on whenever the motion sensor was activated, displaying a neutral image of a flower that changed to a slightly different flower when the dispenser was used (the same logic as in the Screen-Emoticon condition). The changing image was also supposed to act as a reminder for hand hygiene by attracting attention but not activating social norms (BCT-T-v1: prompts/cues).

3) *Picture-Neutral*: In the second neutral control condition, there were no changing screens. Instead, a stationary image of a flower was presented at all times. In the Picture-Neutral condition, neither social norms were activated nor feedback provided (BCT-Tv-1: prompts/cues).

4) *Picture-Eyes*: The setup of the eye condition was identical to the Picture-Neutral condition, except that an image of human eyes was presented. The picture was a visual cue to remind people about hand hygiene, and was intended to make social norms salient through the feeling of being observed. No feedback about hand hygiene status was provided (BCT-Tv1: prompts/cues and social comparison).

All images were displayed in black and white to avoid the influence of any colour. Also, the emotional quality of all pictures was pre-tested in a survey, as has been done in other studies regarding hand hygiene (e.g., [62]). The participants rated to what extent the images elicited different emotions on a five-point scale, from 1 (*not at all*) to 5 (*very much*). The measure of the emotion *guilt* was of special interest, as it was expected to be a suitable proxy for the pictures’ ability to activate social norms in the hospital. The survey data showed that the picture of the frowny and both eye images had significantly higher guilt-ratings than the three other pictures, which we interpreted as being suitable candidates to induce social norms in the experiment (for statistical analysis see S1 Appendix).

### Measures

**Fig 2:**
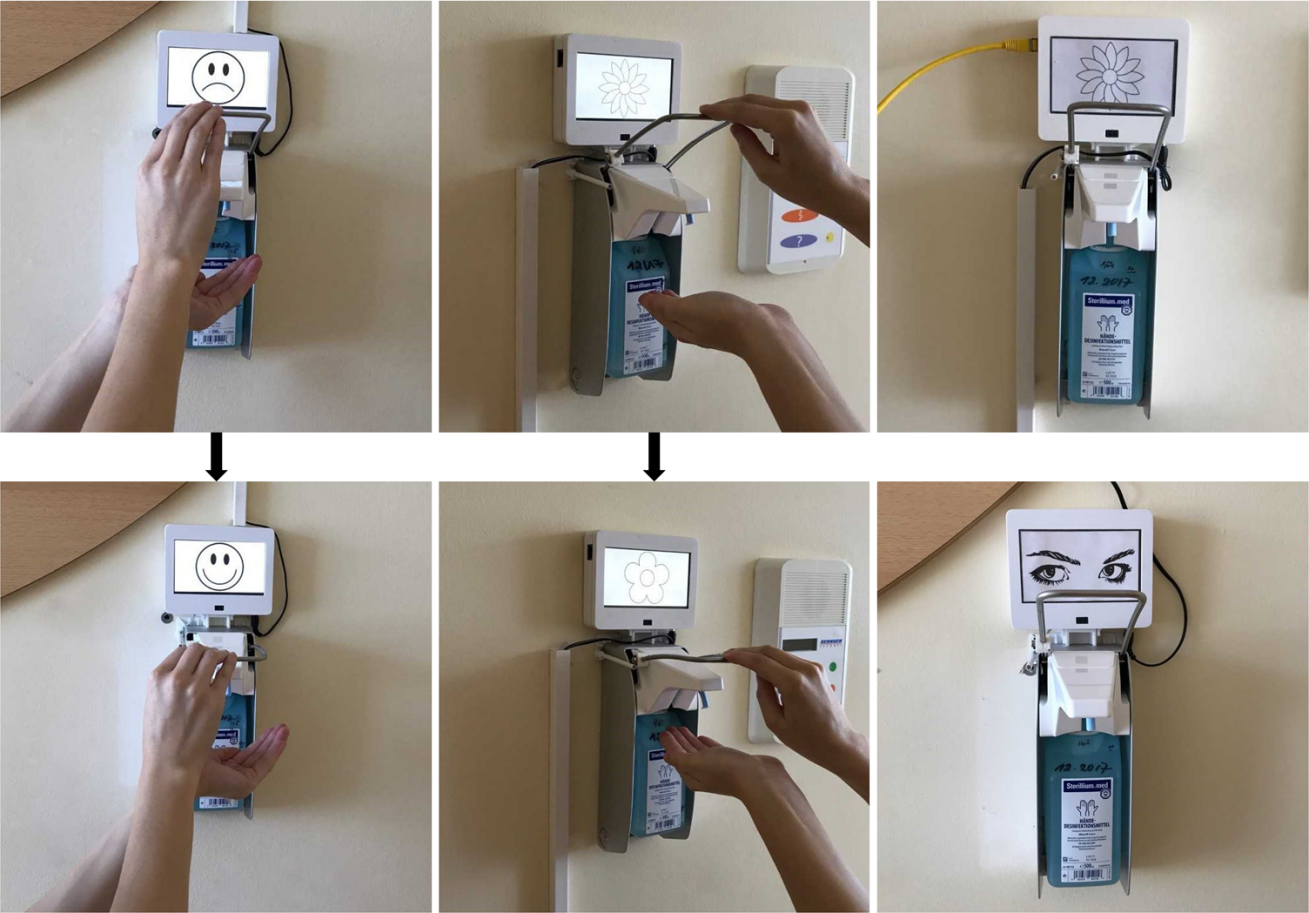
Pictures of the intervention set-up.

The dependent variable was the percentage (ratio) of hand hygiene events (HHE, i.e., using the hand-rub dispenser) among all motion sensor activations, which covers every entrance and exit of the rooms. Electronic devices were installed on top of the existing dispensers to measure room traffic, HHE, and display the images. Those devices (see Fig 2) were white plastic boxes, which included: 1) Infrared motion sensors with a sensor range of 60cm to register people passing by the dispenser; 2) Screens to display the above-mentioned images, or frames for the stationary pictures; 3) Magnetic sensors to measure dispenser usage (i.e., pressing the lever); and 4) Computers to process the information. The monitors and stationary pictures within the devices were five inches (11cm x 6.5cm) in size and in good visual sight from the entrance. However, the overall number of motion sensor activations is not a perfect proxy for room entrance and exit, as everyone who uses the bathroom within a patient room passes the sensor. Thus, the total number of activations also includes the traffic between beds and bathrooms.

The data collected included a time measure, the counts of motion sensor activations (i.e., entering and leaving the patient room or bathroom), and HHE (i.e., usage of dispenser, which counts as HHE independent of how many times the lever was pressed). When the motion sensor was activated, a period of 25 seconds started. In each sensor period, it was measured whether or not a HHE occurred or not. The motion sensor ignored all movements within this period of 25 seconds so that the initial picture could be presented.

### Procedure

Prior to the study set-up, staff and patients were informed about the experiment. The study lasted 17 weeks. In the first eight weeks, the baseline HHE ratio was measured within the eight rooms. During the baseline phase, all screens were off, and there were no images on the devices without screens. A nine-week intervention phase followed immediately afterwards. At the start of the intervention phase, two rooms each, making up the total of eight rooms, were randomly allocated to one of the four conditions (Screen-Emoticons, Screen-Neutral, Picture-Eyes, Picture-Neutral). The images presented on the screens in the *Screen-Emoticons* and Scree-Neutral conditions remained the same during the entire intervention phase. However, the pictures in the conditions with the stationary devices (Picture-Eyes and Picture-Neutral) were changed after the first month of the intervention: Flower 1 was replaced with Flower 2, and Eyes 1 was replaced with Eyes 2. This was done to control for minor differences in the emotional quality of the eye images, and to include both images of the neutral stimuli (Flower 1 and Flower 2) within the stationary devices. The data was collected on regular occasions.

## Results

In total, 65,907 hand hygiene opportunities (motion sensor activations) and 3,340 HHE were registered over the entire study period. The data was arranged to provide one data point with the average of hand hygiene opportunities and HHE for every week. This approach should have led to a total of 17 data points per room–eight at the baseline phase and nine at the intervention phase. However, in the baseline phase, devices in three of the eight rooms encountered technical issues. Data from two weeks are missing in one of the Screen-Neutral rooms and data from six weeks are missing in one of the Screen-Emoticon and one of the Picture-Eyes rooms respectively. In addition, the motion sensor activation data for one room of the Screen-Neutral condition in the intervention phase was not recorded for four weeks, even though all sensors and the screen worked. HHE data was documented without problems. In total, 8.8% of the data were missing. Missing data were replaced using the multiple imputation tool available from SPSS 25 by creating 20 imputed data sets, which has been recommended in the literature [63]. Both motion sensor activations and hand hygiene events over the 17 weeks were included in the imputation model. The average of the pooled results was then taken as the estimate of the missing value for the analysis.

### Hand hygiene event ratio

**Fig 3:**
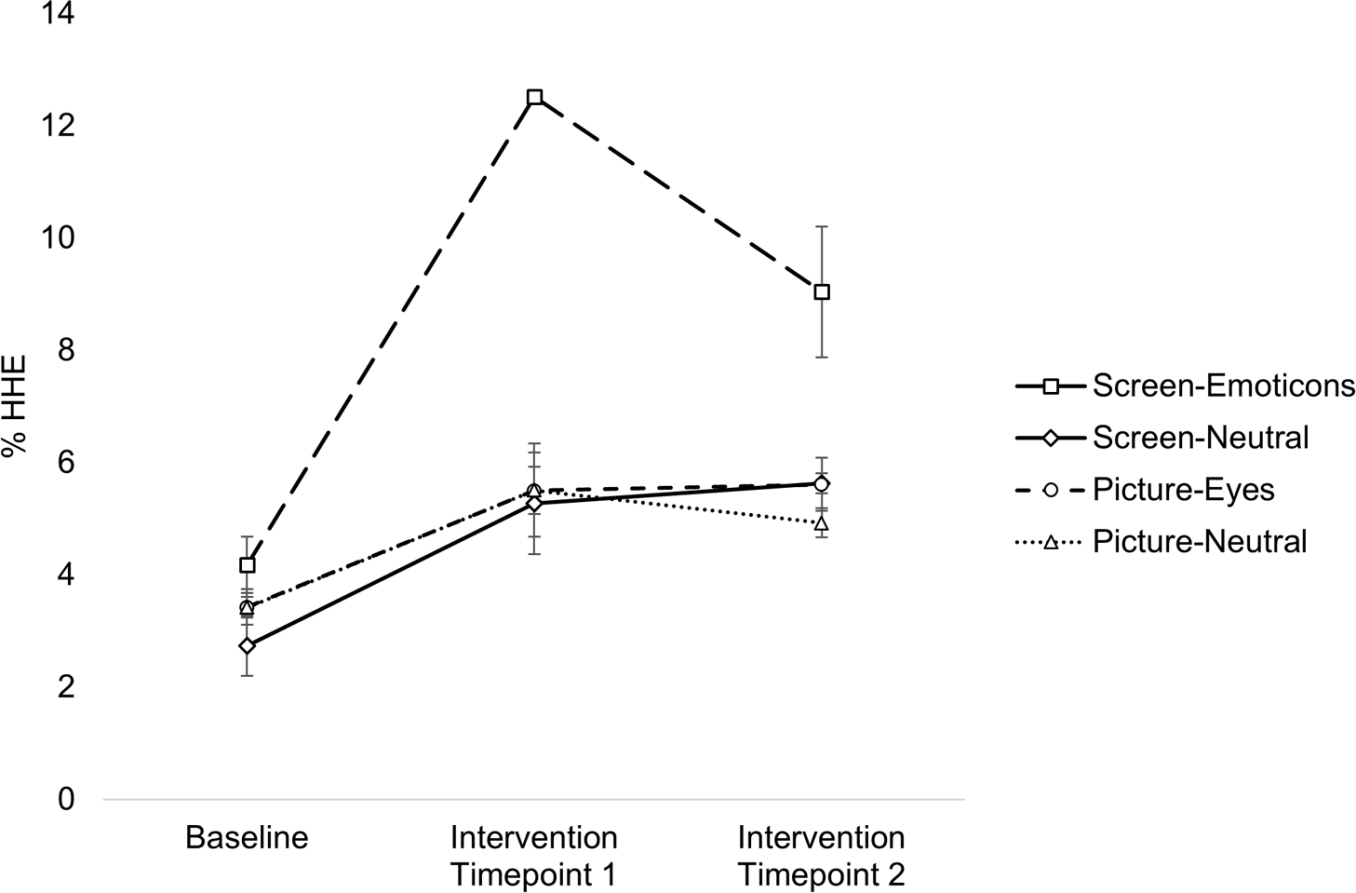
Hand hygiene event ratio data. Mean HHE ratio during baseline phase, intervention phase at intervention timepoint 1 and intervention timepoint 2 for each condition: Screen-Emoticons, Screen-Neutral, Picture-Eyes and Picture-Neutral. Standard errors are presented as error bars.

It was tested whether or not there were any differences between the conditions regarding the dependent variable HHE ratio. HHE ratio per week was calculated by dividing the number of HHE by the number of motion sensor activations, and multiplying by 100 (i.e., calculating the percentage of HHE overall motion sensor activations). The eight weeks of data from the baseline phase were combined to form the baseline measure, and the nine weeks of data from the intervention phase were divided into two timepoints (intervention timepoint 1 included the data from May, and intervention timepoint 2 the data from June) to see if the intervention’s effect was stable over time. The data was clustered in this manner because both room traffic and hand hygiene behaviour are affected by contextual factors on a short-term basis. These factors included fluctuations in room occupancy, patients’ need for care, and seasonal influences such as holidays. While these aspects might bias the hand hygiene event ratio in the short run, they should be randomized out over a longer period. It should be mentioned that there were temporal changes on a weekly basis. However, as mentioned, these fluctuations cannot be interpreted without considering all contextual factors. The HHE data were analysed using a two-way repeated-measures ANOVA with phase (baseline vs intervention timepoint 1 vs intervention timepoint 2) as the within-subjects factor and condition (Screen-Emoticons vs Screen-Neutral vs Picture-Eyes vs Picture-Neutral) as the between-subjects factor. HHE ratio was normally distributed, as assessed by Normal Q-Q Plot. No studentized residual had a value greater than ±3, therefore, this implied that there were no outliers in the data set. The assumption of sphericity was met according to the Mauchly’s test of sphericity, *χ2* = 4.498, *p* = .106. The ANOVA revealed a significant interaction between the phase of the experiment and the four conditions on HHE ratio, *F*(6, 8) = 11.128, *p* = .002, $\eta_{p}^{2}$ = .893. There was also a significant main effect for phase, *F*(2, 8) = 70.721, *p* < .001, $\eta_{p}^{2}$ = .946, and a main effect for condition, *F*(3, 4) = 19.969, *p* = .007, $\eta_{p}^{2}$ = .937. Fig 3. shows the mean ratios for all conditions between the timepoints.

To further investigate whether or not the HHE ratio differed between conditions in the baseline and intervention phase (intervention timepoint 1 and intervention timepoint 2), three separate one-way ANOVAs were conducted. No statistically significant difference in HHE ratio between the four conditions at the baseline phase was found, *F*(3, 4) = 2.065, *p* = .248, $\eta_{p}^{2}$ = .608. Accordingly, the Tukey HSD post hoc analysis found no mean HHE ratio differences between the four groups Screen-Emoticons (*M* = 4.180, *SD* = 0.711), Screen-Neutral (*M* = 2.738, *SD* = 0.755), Picture-Eyes (*M* = 3.429, *SD* = 0.448) and Picture-Neutral (*M* = 3.424, *SD* = 0.265).

There was a significant difference in HHE ratio between the conditions at the first timepoint during the intervention phase, *F*(3, 4) = 29.797, *p* = .003, $\eta_{p}^{2}$ = .957. The post hoc test revealed that the Screen-Emoticon group (*M* = 12.518, *SD* = 0.118) had significantly higher means than Screen-Neutral (*p* = .005), Picture-Eyes (*p* = .005) and Picture-Neutral (*p* = .005). There were no significant differences between Screen-Neutral (*M* = 5.280, *SD* = 1.278), Picture-Eyes (*M* = 5.506, *SD* = 0.592) and Picture-Neutral (*M* = 5.511, *SD* = 1.172). This means that the average HHE ratio at 12.5% in the emoticon condition was statistically higher than in the other three conditions (Screen-Neutral: 5.3%, Picture-Eyes: 5.5% and Picture-Neutral: 5.5%) at intervention timepoint 1.

HHE ratio also varied significantly between the conditions at the second timepoint in the intervention phase, *F*(3, 4) = 8.145, *p* = .035, $\eta_{p}^{2}$ = .859. According to the Tukey HSD test, Screen-Emoticons (*M* = 9.041, *SD* = 1.653) differed significantly from Picture-Neutral (*M* = 4.928, *SD* = 0.367, *p* = .037) and marginally significantly from Screen-Neutral (*M* = 5.634, *SD* = 0.256, *p* = .067) and Picture-Eyes (*M* = 5.613, *SE* = 0.666, *p* = .066), while there was no mean difference between the other conditions. These results mean that the HHE ratio of the Screen-Emoticon group at 9.0% at intervention timepoint 2 was still significantly higher than in the Picture-Neutral rooms at 4.9%, and slightly higher than in the Screen-Neutral at 5.6% and Picture-Eyes at 5.6%.

To test if the HHE ratio differed within the conditions over time, a repeated measures ANOVA over the data, split into conditions, was conducted. There was a significant effect of phase on the HHE ratio for the Screen-Emoticons condition, *F*(2, 2) = 44.702, *p* = .022, $\eta_{p}^{2}$ = .978. The HHE ratio in the Screen-Emoticons group increased from 4.2% at baseline phase to 12.5% at intervention timepoint 1, which is almost a three-fold surge in hand hygiene behaviour. Also, the 9.0% HHE ratio at the second intervention timepoint was still higher than the 4.2% HHE ratio for the baseline measure. At intervention timepoint 2, the dispensers in the emoticon rooms were used more than twice as often as during the baseline measure. However, HHE ratio decreased from 12.5% at intervention timepoint 1 to 9% at intervention timepoint 2. The post hoc analysis did not reveal any significant effects for the Screen-Emoticons condition. A significant simple main effect for phase on HHE ratio was found for the Screen-Neutral condition as well, *F*(2, 2) = 19.109, *p* = .050, $\eta_{p}^{2}$ = .950. The HHE ratio in the Screen-Neutral condition increased from 2.7% at baseline phase to 5.3% at intervention timepoint 1. The HHE ratio remained comparatively stable within the intervention phase (5.2% and 5.6% respectively). Again, the post-hoc analysis did not show any significant results. No significant effect of phase on HHE ratio was found for the Picture-Eyes condition, *F*(2, 2) = 6.703, *p* = .130, $\eta_{p}^{2}$ = .870, or the Picture-Neutral condition, *F*(2, 2) = 9.390, *p* = .096, $\eta_{p}^{2}$ = .904.

## Discussion

In the present study, we explored if targeting social norms improves hand hygiene behaviour in a hospital. The most important finding was that the emoticon-based feedback system significantly increased the usage of hand-rub dispensers in patient rooms, in comparison to the three other tested conditions. This result indicates that the activation of injunctive norms through emoticons, which potentially act as indicators to perform the behaviour to prevent forgetfulness, is an effective approach to improve hand hygiene behaviour.

The primary aim of the data analysis was to investigate the HHE ratio differences between the four different conditions, Screen-Emoticons, Screen-Neutral, Picture-Eyes and Picture-Neutral, over the three phases (baseline, intervention timepoint 1 and timepoint 2). The analysis showed that there were no baseline differences in HHE ratio among the four groups. This implies that prior to the intervention, the rooms in the four conditions did not differ in their usage of the dispensers. This changed after the implementation of the intervention. The dispensers in the rooms with screens displaying a frowny/smiley were used more than twice as often as dispensers in the other patient rooms (i.e., the other three conditions) over the first month after implementation. In the second month, dispensers in the Screen-Emoticons rooms were still used more regularly than the ones in the other three conditions on a descriptive level, but the mean difference was smaller, and only compared to the Picture-Neutral condition was this difference statistically significant.

These results can be used to answer the three postulated research questions. First, in the present experiment, the display of emoticons indeed improved hand hygiene behaviour in the patient rooms. As indicated by previous research [56], it was assumed that emoticons would activate injunctive norms (i.e., ‘I should do, what ought to be done’) by providing instant feedback about a person’s hand hygiene status. The appearance of the frowny face likely acted as an indicator for people that hand hygiene behaviour should occur at this point, and that non-compliance was unacceptable. After using the dispenser, the screen changed from a frowny to a smiley face. This positively reinforced the hand hygiene behaviour performed by showing that this was the desired thing to do. Both aspects might have helped to prevent forgetfulness, which is particularly important for patients and visitors, who receive no formal training. It appears as if injunctive feedback was the main driver for the improvement of HHE ratio in this study. The hand hygiene behaviour in the Screen-Neutral condition did not improve as substantially as in the Screen-Emoticon condition. This suggests that the positive effect in the Screen-Emoticons condition cannot solely be explained through attracting attention via the changing screens. Thus, research question 1 was affirmed in general. However, the positive effect of the emoticon-based feedback system lessened over time. More research is needed to identify the main reason for this decline and find ways to mitigate the reduction in effectiveness.

Presenting eye-like stimuli did not improve hand hygiene behaviour in the patient rooms. Therefore, the watching eyes effect was not found in the present study, and research question 2 must be negated. This is in accordance with other studies, which have also failed to replicate the watching eyes effect (see for instance [49]). It has been suggested that the surveillance-effect might depend on the number of other people present in the situation. For instance, two experiments found no strong positive effect of eye-stimuli on donations when a supermarket was highly frequented with customers [64,65], and two other studies found similar results on littering behaviour ([66,67] but see [47] for an opposing result). In our experiment, there were usually several people present in the patient rooms. If the eye-cue indeed activates a search for references about how other people behave and no other hospital employee, patient or visitor cleans their hands, skipping hand hygiene might be accepted as the appropriate norm. This could be one possible explanation for why the watching eyes effect was not found. A second explanation could be that our images looked rather feminine, and were drawings instead of real photographs. In some previous studies, the watching eyes effect was only found for pictures of sombre looking male eyes (e.g., [52] but see [53] for an opposing result). On the other hand, the watching eye effect has even been found in experiments using so-called *eye spots* [46,64], which are not images of real human eyes but can be interpreted as eyes. A third—and according to the literature increasingly more plausible—explanation would be that eye cues indeed do not notably influence human behaviour [49].

Lastly, it was expected that visual cues displaying emoticons, which potentially activate injunctive norms by providing instant feedback, would improve hand hygiene behaviour more effectively than eye images, which we believe to make descriptive norms salient. The data supported this assumption, and thus, research question 3 can be answered in the affirmative. This result builds on the findings of previous research [56], which also found that targeting injunctive norms was more robust in changing people’s behaviour than descriptive norms.

The present experiment is the first study to test the effect of activating injunctive norms through an emoticon-based feedback system on hand hygiene behaviour in a hospital. To our knowledge, none of the studies investigating the effectiveness of emoticons in traffic–which from a technical standpoint are similar to the present experiment–tested underlying psychological mechanisms. But, it is plausible that the psychological principles are the same. Therefore, the results of this experiment contribute to the theoretical understanding of how injunctive norms might modify behaviour in other research areas as well. Our results add promising and novel findings about the effectiveness of applying injunctive norms in health behaviour change interventions to the literature.

This study had several limitations. First, the sample size of eight patient rooms was small. The limited number of rooms impacted the data analysis because it inhibited the assumptions of homogeneity of variance and equality of covariance. Despite the small sample size, the results could be interpreted due to the strength of the measured effects. Therefore, subsequent studies should include more rooms. Second, some technical problems occurred. As this study was a pilot intervention, technical challenges were to be expected. However, the missing data points had no severe impact on the general experiment. Third, it is difficult to compare the results from this experiment with other interventions to increase hand hygiene behaviour for two reasons. Firstly, in the present study, hospital staff, patients and visitors were not distinguishable. Therefore, it is not possible to infer if the intervention affected each group of people differently. However, most other studies focused only on hospital employees. Secondly, the motion sensor activations were not a perfect proxy for hand hygiene opportunities. One reason for this is that traffic to and from the patients’ bathroom are included in that data. Another reason is that the motion sensors ignored all movements within a 25 second period after activation. If more people entered the room simultaneously, entered and left the room within this period, or lingered right in front of the dispenser, the log file would only show one motion action and connect all HHE occurring within this period to this activation. Therefore, HHE ratios measured in this study must not be misunderstood as a compliance rate, which is usually the dependent measure to evaluate interventions. The final limitation is concerned with the quality of hand hygiene. As with all electronic measures of hand hygiene, no assertion about the quality of hand hygiene behaviour (i.e., if enough hand-rub was used and if it was properly spread over the hands), as well as about the necessity to perform a HHE (e.g., if a nurse entered the room without touching the patient and the patient’s environment), can be made.

In conclusion, this work presents preliminary evidence that inducing injunctive norms via emoticons can improve hand hygiene behaviour in hospitals. The emoticon-based reminders indicated to people that hand hygiene behaviour should be performed via a frowny emoticon. Consequently, a smiley face positively reinforced adequate behaviour (i.e., using the dispenser). Hand hygiene rates in the emoticon-condition significantly outperformed the three other conditions. However, the positive effect seems to be rather temporary. Therefore, future research should be conducted to find optimal ways on how the positive effect of triggering injunctive norms can be employed to sustainably increase hand hygiene compliance in healthcare facilities. These findings might also be applied to other healthcare-related behaviour change settings.

## Supporting information

S1 Dataset Data hospital. SPSS Data of the experiment in the hospital.
(SAV)

S2 Dataset Data online survey. SPSS Data of the online survey.
(SAV)

S1 Appendix Results of the online survey. (DOCX)
