# Supplementary material for: How a smiley protects health: A pilot intervention to improve hand hygiene in hospitals by activating injunctive norms through emoticons
Source: PLoS One. 2018 May 21;13(5):e0197465. doi: 10.1371/journal.pone.0197465 (PMC5962087; doi:10.1371/journal.pone.0197465)
Supplement: S1 Appendix — (DOCX) [file pone.0197465.s003.docx]

**S1 Appendix. Results of the online survey.**

### Measures & Analysis

To measure the emotional quality of the images, the six pictures were shown to 36 participants in an online survey. The participants rated to what extent the images elicited different emotions (i.e., interest, joy, surprise, sadness, anger disgust, contempt, fear, shame and guilt; based on Izard’s (1972) model of emotions) on a five-point scale, from 1 (*not at all*) to 5 (*very much*). The *guilt* measure was of special interest as a proxy for social norms. To test for differences in emotional quality between the six images, Friedman tests and post-hoc tests were conducted. For the analysis, the questionnaire scale was inverted so higher scores represented higher emotion ratings.

# **Results**

The Friedman test results showed significant differences in the emotion ratings (all emotions combined) between the six pictures χ^2^(5) = 50.521, p < .0005. Pairwise comparisons were performed with a Bonferroni correction for multiple comparisons. Emotion ratings were significantly higher for Eyes 1 (*M* = 2.32, *SD* = 0.67), compared to Flower 1 (*M* = 1.73, *SD* = 0.40, *p* < .001), Flower 2 (*M* = 1.66, *SD* = 0.47, *p* < .001), and Smiley (*M* = 1.84, *SD* = 0.49, *p* = .003). The emotion level of Eyes 2 (*M* = 2.15, *SD* = 0.7) surpassed the ratings for Flower 1 (*p* = .042) and Flower 2 (*p* < .001). Finally, the emotion scores for the image of the Frowny (*M* = 2.04, *SD* = 0.71) were higher than the ratings of Flower 2 (*p* = .001). All other images did not differ significantly in their emotion-level ratings.

When comparing the pictures on their scores on the emotion *guilt*, there was also a significant difference between the six pictures, χ^2^(5) = 56.636, p < .001. The Bonferroni corrected pairwise comparison showed that Eyes 1 (*M* = 2.08, *SD* = 1.16) had higher guilt ratings than Flower 1 (*M* = 1.14, *SD* = 0.42, *p* = .015), Flower 2 (*M* = 1.19, *SD* = 0.71, *p* = .029), and Smiley (*M* = 1.17, *SD* = 0.61, *p* =.036). The guilt level for the image of Eyes 2 (*M* = 2.14, *SD* = 1.38) exceeded the level for Flower 1 (*p* = .032). The picture of the Frowny *(M* = 2.14, *SD* = 1.38) also produced significantly higher guilt ratings than Flower 1 (*p* = .023) and Flower 2 (*p* = .045). No other comparisons reached statistical significance. Mean guilt ratings are displayed in Fig 1.

**Fig 1.** Mean guilt ratings of the presented stimuli. Standard errors are presented as error bars.
